# A systematic appraisal of the information, engagement, aesthetic and functional quality of nutrition-related smartphone apps for children and adolescents

**DOI:** 10.1017/S1368980023000526

**Published:** 2023-07

**Authors:** Lucine Francis, Erin M Spaulding, India Bloom, Alisha Patel, Nancy Perrin

**Affiliations:** 1 Center for Community Programs, Innovation, and Scholarship, Johns Hopkins University School of Nursing, Baltimore, MD 21205, USA; 2 Johns Hopkins University School of Nursing, Baltimore, MD 21205, USA; 3 Digital Health Innovation Laboratory, Ciccarone Center for the Prevention of Cardiovascular Disease, Division of Cardiology, Department of Medicine, Johns Hopkins University School of Medicine, The Welch Center for Prevention, Epidemiology and Clinical Research, Johns Hopkins University Bloomberg School of Public Health, Baltimore, MD 21205, USA

**Keywords:** Nutrition Apps, mobile App Rating, quality, smartphone, mobile applications, children

## Abstract

**Objective:**

Nutrition-related smartphone applications (apps) could improve children’s nutrition knowledge and skills. However, little is known about the quality of nutrition-related apps for children. This study aimed to identify and evaluate the quality of nutrition-related smartphone apps designed for children ages 4–17.

**Design:**

This systematic appraisal is guided by the Protocol for App Store Systematic Reviews.

**Setting:**

Using Google’s Advanced Search, we identified 1814 apps/1184 additional apps in an updated search on iOS, of which twenty-four were eligible. The apps’ objective and subjective quality were evaluated using the twenty-three-item, five-point Mobile App Rating Scale. The objective quality scale consists of four subscales: engagement, functionality, aesthetics and information.

**Results:**

Most of the apps (75 %) focussed solely on promoting nutrition skills, such as making food dishes, rather than nutrition knowledge. Of the twenty-four apps, 83 % targeted children 4–8 years old. The app objective quality mean score was 3·60 ± 0·41. The subscale mean scores were 3·20 ± 0·41 for engagement, 4·24 ± 0·47 for functionality, 4·03 ± 0·51 for aesthetics and 2·94 ± 0·62 for information. The app subjective quality mean score was 2·10 ± 0·90.

**Conclusions:**

More robust approaches to app development leveraging co-design approaches, including involving a multidisciplinary team of experts to provide evidence-based nutrition information, are warranted.

Obesity affects nearly one in five children and adolescents in the USA, with Black and Hispanic communities most affected^(1,2)^. Although the causes of childhood obesity are complex, poor nutrition contributes significantly to obesity in children and adolescents^(3–5)^. Childhood obesity can lead to a lifetime of health issues such as type 2 diabetes, high blood pressure, sleep disorders, asthma and social problems such as being bullied^(4,6)^. Settings where children spend the most time, like childcare and schools, can be leveraged to provide optimal nutrition education and implement evidence-based obesity prevention interventions. Evidence suggests that lockdowns such as the closures of schools and childcare related to the COVID-19 pandemic have exacerbated existing disparities in childhood obesity, especially for households in low-income and minoritized communities^(7)^. To add, pandemic-related changes in children’s eating behaviours and physical activity mirror weight gain patterns experienced during breaks from school when access to healthy foods and opportunities for physical activity are often limited^(8–11)^.

Given the magnitude of the obesity epidemic in the USA, there is a need for cost-effective, accessible and low-touch solutions to promote healthy nutrition among children and adolescents. The use of high-quality mobile health (mHealth) applications (apps) represents one promising avenue for health promotion, with mHealth apps exploding in popularity over the past several years. Although more experimental research is needed, some evidence suggests that nutrition-based apps are helpful, especially for promoting fruit and vegetable consumption among children^(12–14)^. Sixty percent of parents in the USA with children 12 years or younger reported that their children engage with smartphones, with 30 % doing so before age 5^(15)^. Most US teens aged 13–17 years have access to a smartphone, and more than half report using it to *‘pass time,’* meet others and for learning purposes^(16)^. With the increased use of smartphones among children and adolescents, engaging with nutrition-related mobile apps presents an opportunity for health promotion centred on nutrition. Already, parents and teachers rely on educational apps and game-based learning to support the mastery of literacy and math skills^(17)^. Likewise, nutrition education in US schools is prevalent throughout the K-12 experience, but the frequency of the teaching and the quality of the curricula are not fully understood^(18,19)^. Mobile apps can help build a widely accessible and high-quality digital nutrition learning community to improve nutrition knowledge and skills and help to prevent obesity.

Prior research has demonstrated the potential for mHealth apps to aid in weight loss and improve dietary behaviours, mainly in adults^(20–22)^. Few studies have focussed on identifying currently available health promotion apps explicitly designed to encourage healthy nutrition knowledge and practices, especially in children and adolescents. Little is known about the existence, content and quality of nutrition-related apps currently available in smartphone app stores, particularly those directed towards children and adolescents^(23)^. In a recent review, the quality of nutrition promotion apps and websites was systematically appraised to assess their usability and effectiveness for improving child nutrition^(24)^. However, the study focussed on appraising digital tools directed towards parents to help support their children’s nutrition. More evidence is needed to identify and examine the quality of nutrition-related apps designed for children and adolescents to be the end-user.

This systematic appraisal aims to identify and appraise nutrition-related smartphone apps’ information, engagement, aesthetic and functional quality for children ages 4–17. We aim to ask the following research questions:

1. What free nutrition-related apps in the English language exist for children?
2. What is the app’s quality concerning the information, engagement, aesthetic and functionality?
3. Have the identified apps been studied extensively to examine their impact on child nutrition or health outcomes and findings published in the peer-reviewed literature?

## Methods

### Study design

We conducted the systematic search, appraisal and reporting of nutrition-related apps for children according to the Protocol for App Store Systematic Reviews (PASSR),^(25)^ which was adapted from the A MeaSurement Tool to Assess Systematic Reviews (AMSTAR)^(26)^ and the Preferred Reporting Items for Systematic Reviews and Meta-Analyses (PRISMA)^(27)^.

### App search strategy

Our search strategy was guided by the PICOS model included in PASSR; **P**opulation: children including adolescents (4–17 years old); **I**ntervention: nutrition-related apps; **C**ontrol: none; **O**utcomes: ratings of app’s information, engagement, aesthetics and functional quality; **S**tudy design: a cross-sectional appraisal. We used Google Advanced Search^(28)^ to search for apps within the Apple store (itunes.apple.com) by using the following terms: ‘nutrition AND kids,’ OR ‘foods AND kids,’ OR ‘beverages AND kids,’ OR ‘cooking AND kids,’ OR ‘obesity AND kids,’ OR ‘weight AND kids.’ We used Google Advanced Search because it allows users to apply filters (such as ‘all of these words’ or ‘any combination of these words’) that enhanced the quality of our search. This approach has also been used by other researchers conducting ratings of mobile apps^(29–31)^. We searched only within the Apple app store to reduce bias in this way: Apps within the Apple app store are generally of higher quality than Google Play apps because the approval process for Google Play is much faster and less strict^(32)^. Therefore, to reduce bias, we chose to conduct the review solely within the Apple app store, which also provides the advantage of being able to search for free apps. We restricted the search to results that included ‘all these words’ and ‘English language only,’ meaning that the app store page was available in English so that reviewers could evaluate app descriptions and other relevant information displayed on the app store page. Links from each Google Search page were extracted using a link scraping tool called Linkclump^(33)^ and then pasted into Excel^(34)^. The final search for this review was conducted on October 26, 2020, and an updated search since the initial search was conducted on August 5, 2022.

All duplicate apps were removed from the Excel sheet using Excel’s sort function and the ‘ctrl+F’ function. If available, we only included the English language version for apps with multiple language versions. We also considered apps duplicates if they were older versions of an app or had slightly different artwork but otherwise had identical descriptions and names. iOS links that were either entirely unavailable or not available in the US app store were also removed. A master list of links was created after removing duplicates and unavailable links.

### Eligibility criteria and screening process

#### Pre-installation screening process

Apps were eligible for inclusion in the appraisal if they met the following criteria: (1) English language only, (2) free to download and included no in-app purchases, (3) designed with children (aged 4–17 years) in mind as the end-user and (4) contained food or beverage-related content. Regarding eligibility criteria 2, free apps in Apple’s App store are more likely to rank in the top 300 grossing apps and have a two to three times higher survival rate than paid apps^(35)^. The rationale for including only free apps in this review was to identify apps that could be used widely without barriers related to costs since App cost is cited as a primary concern for mHealth app users^(36)^. Regarding the third eligibility criteria, apps were designated as eligible if the Apple app store rated the app as for ages 4+ and the app was not targeted at professional or solely adult audiences (e.g. apps for healthcare professionals or parenting apps). The iOS app age ratings are 4+, 9+, 12+ and 17+. A rating of 4 + means the app contains no objectionable material, but not necessarily mean that the app is designed for children^(37)^. A rating of 9+, 12+ and 17 + means the app may contain content that may not suit children under those age groups. Apps were excluded if they: (1) were designed for adult use only; (2) were deemed inappropriate for children due to profanity, nudity, alcohol/drug use, violence or crude humour or (3) contained no nutrition or food/beverage content per the description in the iOS app store. Two independent reviewers (ES and IB) assessed each app description in the iOS app store for eligibility. Eligibility disagreements were resolved with a third reviewer (AP).

#### Post-installation screening process

The apps that met the eligibility criteria during the pre-installation screening were downloaded onto two independent reviewers’ (ES and IB) iPhones. Eligibility for inclusion was then confirmed upon engaging with the apps. In addition to the aforementioned eligibility criteria, the apps were also excluded at this stage if they solely contained obesogenic or obesity-promoting foods. We excluded these apps to focus on nutrition-related apps that could be considered health promotion tools among school aged children. An obesogenic screening tool was developed for this purpose by the study team. The obesogenic screening tool asked, ‘Does this app **only** contain foods that are top sources of added sugars, saturated fat, or sodium?’ If the answer to this question was ‘yes,’ the app was excluded, and if ‘no,’ the app was included in the review. The criteria used to determine whether foods were obesogenic were mainly developed from data provided by the United States Department of Agriculture (USDA) Food Surveys Research Group^(38–44)^. The mission of the Food Surveys Research Group is to *‘monitor and assess food consumption and related behavior of the US population by conducting surveys and providing the resulting information for food and nutrition-related programs and public policy decisions.’* A list of the foods deemed to be top sources of added sugars, saturated fat or Na can be found in Supplementary Methods 1. During this post-installation phase, the two independent reviewers (ES and IB) assessed each app for eligibility, including using the obesogenic screener. A third reviewer was not required to resolve discrepancies.

### Measures

All data amassed in the appraisal process were documented in Qualtrics^(45)^.

### Demographic data

Two independent reviewers (ES and IB) extracted demographic information for each app included in the appraisal by (1) reviewing the app’s description in the iOS app store and (2) obtaining relevant demographic information by engaging with the apps. The demographic data extracted from the app’s description in the app store included: app name, developer, date of last app update, current version of the app, size of the app in Megabytes (MB), number of users that rated the app, the user rating in the app store (scale of 1 to 5 stars), availability on iPhone and/or iPad and iOS app age rating. The demographic data extracted after interacting with each app included: developer affiliation; whether the app includes ads; the perceived targeted age group; the technical aspects and theoretical strategies incorporated into the app; whether the app aimed to promote nutrition knowledge, skills or both and the areas the app targeted. The reviewers determined the perceived targeted age group by assessing the app’s readability, design and content. For example, apps rated as appropriate for children 4 to 8 years old (pre-kindergarten – 2nd grade) primarily contained pictures but may have had some simple sentences or audio for text (readability), simple/single taps on the iPhone screen (design), minimal pages (design) and easy to understand nutrition information (content)^(46)^. After extracting the data independently, the two reviewers (ES and IB) resolved any discordances.

### Quality appraisal using the mobile app rating scale

Two independent reviewers (ES and IB) engaged with the final list of apps for the systematic appraisal for three days using their iPhones. After the third day, they independently assessed the apps’ engagement (i.e. entertainment, customisation, interactivity, fit to target group), information (i.e. quality, quantity, credibility, goals), aesthetic (i.e. graphics, layout, visual appeal) and functional (i.e. performance, navigation, gestural design, ease of use) quality, as well as the apps’ subjective quality using the twenty-three-item Mobile App Rating Scale (MARS)^(47)^. The MARS has demonstrated excellent internal consistency (alpha = 0·90) and interrater reliability (intraclass correlation coefficient (ICC) = 0·79)^(47)^. Before evaluating each app using the MARS, the independent reviewers watched the YouTube training video provided by the MARS developers^(48)^. In addition to the MARS response criteria, the two reviewers developed and used a guide (Supplementary Methods 2) to standardise their responses and improve their interrater reliability.

The MARS objective quality scale (nineteen items) consists of the following subscales: engagement (five items), functionality (four items), aesthetics (three items) and information (seven items). The MARS subjective quality scale consists of four items in which the raters responded to whether they would recommend the app to people who would benefit from it, use the app and frequency in doing so for the next 12 months, pay for the app and provide an overall rating of the app. Other than the item regarding whether one would pay for the app (Yes, No Maybe), all other items have responses ranging from 1 (indicating lower quality (i.e. inadequate)) to 5 (indicating higher quality (i.e. excellent)), a few items in the information subscale have a ‘N/A’ response option. To evaluate whether the apps had been trialled or tested and published in the scientific literature (a MARS item in the information subscale), three independent reviewers (ES, IB and LF) searched Google Scholar and PubMed for the app names.

### Statistical analyses

The apps’ demographic data were summarised using frequencies (percentages) for categorical variables and medians (interquartile ranges) for continuous variables. We calculated the mean score for each MARS objective subscale and the mean score for each overall objective and subjective scale. Per the MARS instructions, questions rated as ‘N/A’ were removed from the mean score calculations. The mean scores were calculated for each rater (ES and IB), and then the average of the two mean scores were recorded as the final value.

Interrater reliability was initially calculated using the first thirteen appraised apps, but the ICC for the MARS items were poor. Thus, ES, IB and LF developed the guide outlined in Supplementary Methods 2 to improve interrater reliability. After using the guide to rerate the first thirteen apps and then the additional eleven apps (for a total of twenty-four apps), the interrater reliability for the overall MARS objective and subjective quality scale scores were excellent ((ICC = 0·92), (ICC = 0·93)), respectively. The ICC for the MARS objective quality subscales were as follows: engagement (ICC = 0·88), functionality (ICC = 0·79), aesthetics (ICC = 0·72) and information (ICC = 0·85).

## Results

### Search results

**Fig. 1:**
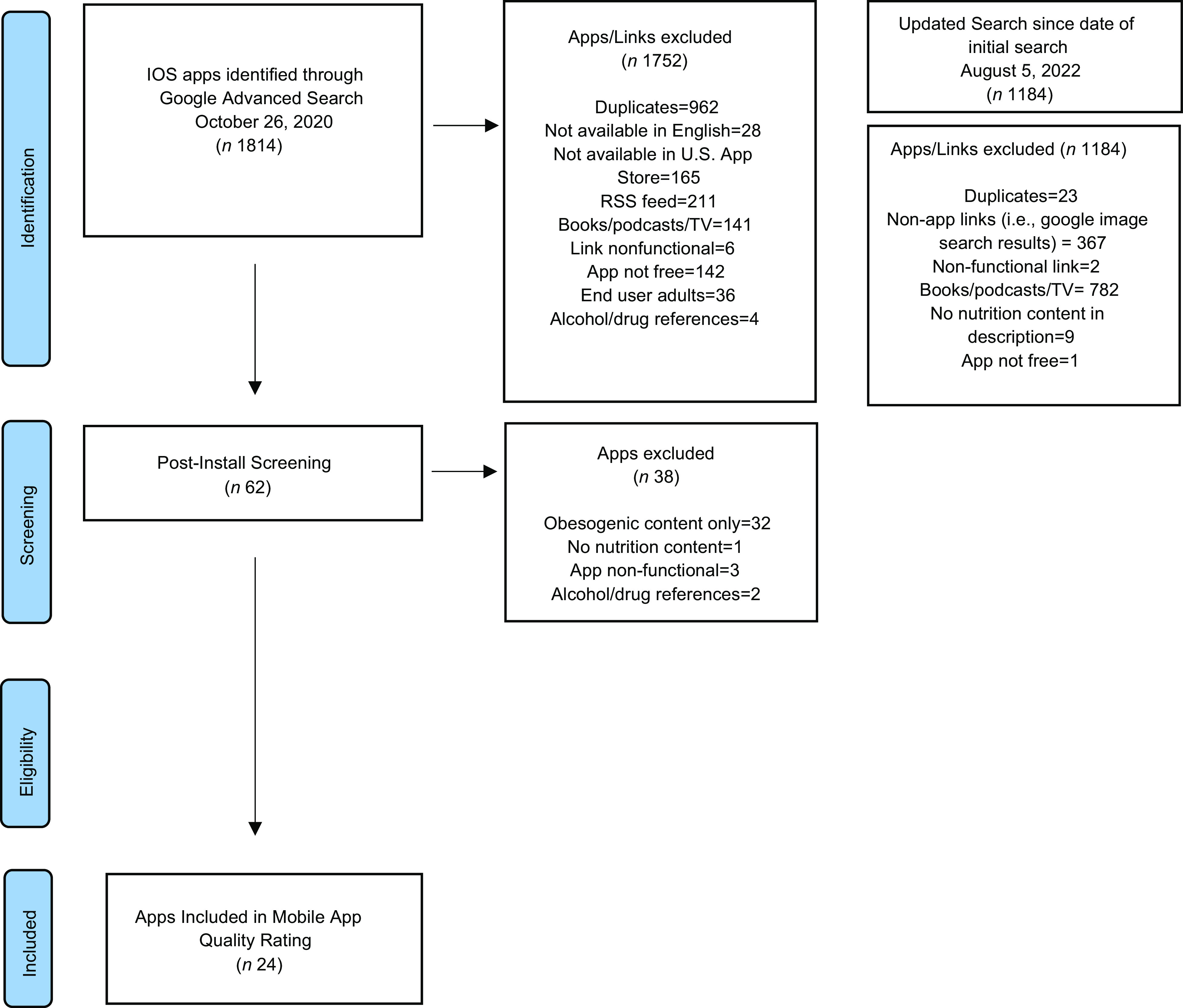
Protocol for App Store Systematic Reviews Diagram Depicting the Flow of Apps

A summary of the search and appraisal process is shown in the Fig. 1. In our original search, a total of 1814 links were identified through Google Advanced Search. In our updated search since the original search, 1184 links were identified. After removing duplicates, pre-install screening was used to determine whether the apps met the eligibility criteria. After the pre-install screening, sixty-two apps were downloaded to conduct post-install screening. Thirty-eight apps were excluded due to having primarily obesogenic content (*n* 32), having no nutrition content (*n* 1), being nonfunctional (*n* 3) and having references to alcohol or drugs (*n* 2). A total of twenty-four apps were included in this systematic appraisal of apps, and no new apps were identified in the updated search that met the eligibility criteria^(49–72)^.

### App characteristics

Per eligibility criteria, all the apps (*n* 24) were designed for children as end-users, in English, and had food or beverage-related nutrition content that was not solely obesogenic. All the apps were rated as appropriate for individuals 4+ years in the iOS app store, indicating that the apps contained no objectionable material. Additionally, all the apps were available for download on iPhones and iPads. The reviewers determined, based on readability, design and content, that 83 % (20/24) of the apps were appropriate for children 4 to 8 years old (pre-kindergarten – 2nd grade), 33 % (8/24) were suitable for children 9 to 11 years old (3rd–5th grade) and 8 % (2/24) were appropriate for children 12 to 16 years old (6th–10th grade). Only one app was updated within the past year at the installation time. The average size of each app was 77·35 megabytes (MB) (range: 13·5 MB–198·1 MB). The median star rating in the iOS app store was 4 (IQR: 2); however, only two of the apps had more than five users who had provided a star rating. Additionally, 87·5 % (21/24) of the apps were developed by an unknown entity since a developer name was not mentioned on the app page; 71 % (17/24) contained ads; and 75 % (18/24) promoted nutrition skills (i.e. cooking, baking), 13 % (3/24) nutrition knowledge (i.e. naming of fruits and vegetables) and 13 % (3/24) both skills and knowledge. None of the apps have been evaluated for effectiveness on user outcomes, as evidenced by a lack of reports in the peer-reviewed literature.

None of the apps contained any technical features noted in the MARS, including allowing for sharing (i.e. Facebook, Twitter), password protection, requiring log-in, sending reminders or needing web access. However, one app did have an app community/leaderboard. The areas most frequently targeted by the apps included (1) entertainment (i.e. gamification; 96 % (23/24)), (2) skill-building (88 % (21/24)) and (3) food/nutrition knowledge or literacy (21 % (5/24)). The theoretical/background strategies most frequently incorporated into the apps included (1) advice/tips/strategies/skills training (96 % (23/24)) and information/education (25 % (6/24)). A summary of the app characteristics can be found in Table 1, and a description of the individual app characteristics in Table 2.

**Table 1:** Summary of App characteristics

	*n* 24	
App Characteristics	*n*	%
iOS App store star rating*		
Median	4	
IQR	2	
Reviewer perceived targeted age group†		
4–8 yrs (PK-2nd grade)	20	83·3
9–11 yrs (3rd–5th grade)	8	33·3
12–16 yrs (6th–10th grade)	2	8·3
Affiliation		
Unknown	21	87·5
Commercial	2	8·3
Non-governmental Organization	1	4·2
Government	0	0
University	0	0
Updated in past year		
Yes	1	4·2
No	19	79·2
Unknown	4	16·7
Contains Ads		
Yes	17	70·8
No	7	29·2
Promoted nutrition		
Knowledge	3	12·5
Skills	18	75·0
Both	3	12·5
Targeted areas†,‡		
Entertainment (i.e. gamification)	23	95·8
Food/nutrition knowledge or Literacy	5	20·8
Skill building	21	87·5
Health and wellness	1	4·2
Theoretical/background strategies†,§		
Assessment	1	4·2
Feedback	2	8·3
Information/education	6	25·0
Goal setting	1	4·2
Advice/tips/strategies/skills training	23	95·8

IQR: interquartile range; yrs.: years; PK: pre-kindergarten.

* Median iOS app store star rating out of twenty-one apps with available data.

† Select all that apply, percentages add up to over 100 %.

‡ The following targeted areas, included both in the MARS or added by the study team, were not reflected in any of the apps and were not included in the table: increase happiness/well-being, mindfulness/meditation/relaxation, reduce negative emotions, depression, anxiety/stress, anger, behavior change, alcohol/substance use, relationships, physical health, skill building, goal setting and weight management.

§ The following theoretical/background strategies, included in the MARS, were not reflected in any of the apps and were not included in the table: monitoring/tracking, CBT-Behavioral (positive events), CBT-Cognitive (thought challenging), ACT-Acceptance commitment therapy, mindfulness/meditation, relaxation, gratitude and strengths-based.

**Table 2:** Individual App characteristics and overall and Subscale mobile App rating scores (*n* 24)

App Name	Developer	Last update	Rating version	Size (MB)	User rating in app store*	App objective quality mean score†	Engagement mean subscore	Functionality mean subscore	Aesthetics mean subscore	Information mean subscore	App subjective quality mean score‡
Baked Lasagna Chef Kids Cooking Game^(49)^	Qamar Zaman	2/4/17	1·0	68·2	N/A§	3·38	3·00	4·25	3·50	2·75	1·50
Chef Cooking – Baby Cotton Candy Cooking Making & Dessert Make Games for Kids^(50)^	Anchalee Pradissook	7/13/15	1·1	57·5	3·7	2·90	3·20	3·63	3·17	1·63	2·00
Cooking Breakfast Maker^(51)^	Amit Gadhiya	7/14/17	1·0·1	15	3·0	3·30	3·00	3·88	4·00	2·33	1·63
Cooking Court Food Fever^(52)^	Kamran Haider	N/A	N/A	176·4	N/A	3·07	3·00	3·00	3·67	2·63	2·00
Cooking Girl, Amy and Cooking Kids Game^(53)^	Wei Li	1/3/17	2·0	16·8	5·0	3·53	3·10	4·50	4·00	2·50	1·38
Cooking Time 2 – Sushi Make & Preschool Kids Game^(54)^	The First Word	3/27/17	6·5	22·2	2·0	3·66	3·30	4·63	3·83	2·88	2·00
Feed Twip^(55)^	Gamabilis	10/3/20	2·4·0	144·1	5·0	4·45	4·40	4·75	4·67	4·00	4·88
FoodLeap^(56)^	National Restaurant Association	3/9/16	1·0·1	15·3	5·0	4·54	4·20	4·88	4·67	4·42	3·75
Fresh Salad Bar: Healthy Green Food Making Game for Education & Learning^(57)^	Kashif Mumtaz	6/3/16	1·0	61·6	N/A	3·56	3·20	3·88	4·17	3·00	2·00
Health and Nutrition Quiz for Kids^(58)^	Kok Leong Tan	9/2/15	1·0	40·2	4·5	3·39	3·20	4·38	2·67	3·30	1·75
Hot Soup Maker – Crazy Chef with Health Food Kitchen Adventure Spicy Cooking Fever^(59)^	Qamar Zaman	7/6/15	1·0	95	3·0	3·33	2·70	4·00	4·00	2·63	1·88
Ice Candy Fever Cooking Game – Cool Kids Food Chef^(60)^	Sohail Jelani	4/15/17	1·0·1	77·2	5·0	3·78	3·20	4·50	4·17	3·25	1·75
Ice Coffee Maker- Make Creamy Dessert in this Cooking Fever Game for kids^(61)^	Hassan Fareed	1/11/16	1·0	85	1·0	3·09	2·30	4·25	3·50	2·29	1·25
Kid Cooking Food: The Funny Restaurant Simulator Free Games^(62)^	Somkiat Kiatpattaranan	7/24/15	1·0	63·6	5·0	3·38	3·10	4·25	3·50	2·67	1·63
Kitchen Kids Cooking Chef: Let’s Cook the most Delicious Food^(63)^	Stefano Frassi	N/A	N/A	167·9	3·0	3·70	3·40	3·88	4·50	3·04	2·00
Mr J Cooks Food, Free Cooking Kids Game^(64)^	令静 孔	N/A	N/A	14·1	5·0	3·60	3·10	4·38	4·17	2·75	1·75
Noodle Maker – Crazy Cooking Adventure for Little Kids Chef Master^(65)^	Qamar Zaman	6/24/15	1·0	54·5	4·0	3·59	3·10	4·38	4·00	2·88	1·88
Pasta Maker Kids Cook – Free Crazy Star Chef Adventure Girls Kitchen Cooking Games^(66)^	Agha Awais Ali Khan	7/9/16	1·0·1	198·1	4·3	3·60	3·20	4·13	4·33	2·75	1·75
Pasta Maker – Kitchen Cooking Chef and Fast Food Game^(67)^	Kids Fun Plus	4/13/16	1·0·2	103	3·6	3·47	3·00	4·50	4·00	2·38	1·75
Sky Burger Maker Cooking Fever – Kids Games^(68)^	Qamar Zaman	11/18/16	1·0·1	145·7	1·0	3·34	3·00	3·38	4·00	3·00	1·50
Spaghetti Maker – Little Kids Cook Chinese Food in this Cooking Fever Game^(69)^	Hassan Fareed	1/21/16	1·0	79·6	3·0	3·70	3·10	4·25	4·50	2·96	1·75
Street Food Cooking Mania – Fun Kitchen Management^(70)^	Kashif Mahmood	N/A	N/A	106	1·5	3·73	3·20	4·50	4·33	2·88	1·63
Veggie Bottoms^(71)^	Red Card Studios LLC	11/19/16	2·0·0	35·8	4·2	4·27	3·50	5·00	4·83	3·75	4·13
Zucchini Spaghetti Bolognese- Vegan Cooking Recipe with Emma: Game for Kids^(72)^	Famobi	1/15/16	1·1	13·5	4·2	4·08	3·20	4·63	4·50	4·00	2·88
Average scale and subscale scores						3·60 (sd: 0·41)	3·20 (sd: 0·41)	4·24 (sd: 0·47)	4·03 (sd: 0·51)	2·94 (sd: 0·62)	2·10 (sd: 0·90)

* Star rating in the app store on a scale of 1.0 to 5.0; all apps were rated by less than or equal to 5 users, except for the ‘Pasta Maker Kids Cook – Free Crazy Star Chef Adventure Girls Kitchen Cooking Games’ app which was rated by 7 users and the ‘Pasta Maker – Kitchen Cooking Chef and Fast Food Game’ app which was rated by 10 users.

† MARS objective rating overall scale score, the average of the two individual raters’ scores for each app; App objective quality mean scores are the average of Engagement, Functionality, Aesthetics, and Information subscores.

‡ MARS subjective rating overall scale score, the average of the two individual raters’ scores for each app; Subjective quality (recommending app, intention to use app and frequency in doing so for the next 12 months, if one would pay for the app, and overall rating of the app).

§ N/A indicates one of the following: (1) no users have rated the app; thus, there is no star rating in the app store or (2) the app has not been updated and there is no last update date or version of the app listed in the app store.

### Mobile app rating scale results

The apps’ objective quality mean score across the 24 apps was 3·60 (sd: 0·41), where a mean score of 1 indicates ‘inadequate’ and 5 indicates ‘excellent’ quality. The objective quality mean score comprises the four subscales mean scores: engagement, functionality, aesthetics and information. The information subscale had the lowest mean score with a rating of 2·94 (sd: 0·62), indicating that the apps did not contain high-quality information from credible sources. The engagement subscale mean score was 3·20 (sd: 0·41), indicating that the apps were only ‘adequate’ in terms of being *fun, engaging, customizable, interactive, and well-targeted to the audience (as defined by MARS)* ^(47)^. The functionality and aesthetics mean scores were higher at 4·24 (sd: 0·47) and 4·03 (sd: 0·51), respectively. Finally, the apps’ subjective quality mean score was rated as 2·10 (sd: 0·90) by the two reviewers, where a mean score of 1 indicates ‘inadequate’ and 5 indicates ‘excellent’ quality. Table 2 provides the objective, subjective scale mean scores and subscale mean scores for each app and all the apps combined.

### App food categories

More than 50 % of the apps included foods in each of the following *What We Eat in America Food Categories: 2017–2018*: protein foods (79 % (19/24)), mixed dishes (54 % (13/24)), grains (63 % (15/24)), fruits (50 % (12/24)), vegetables (92 % (22/24)) and condiments and sauces (63 % (15/24)). Less than 50 % of the apps included foods in each of the following categories: milk and dairy (42 % (10/24)), snacks and sweets (13 % (3/24)), beverages (46 % (11/24)), water (17 % (4/24)), fats and oils (46 % (11/24)) and sugars (13 % (3/24)). We provided the 2017–2018 What We Eat in America food Categories represented in each app as a supplemental table (online Supplementary Table).

## Discussion

This review aimed to identify free nutrition-related mobile apps in English and evaluate their information, engagement, aesthetic and functional quality using the MARS. Only twenty-four free nutrition-based apps for children were identified for this review. Most of the apps in this review were designed for children ages 4–8. With obesity increasing in prevalence as children age, there is a significant need for a wide variety of nutrition-based apps to meet the lifespan needs from early childhood to adolescents to promote healthy nutrition^(1)^. Previous literature has indicated that mobile apps for children can be an effective avenue to enhance diet quality, increase physical activity and reduce BMI in children^(73,74)^. Systematic reviews and meta-analyses of gamified nutrition interventions have demonstrated improvement in important nutritional outcomes, such as the selection of lower-calorie foods, knowledge of healthy foods and increased fruit and vegetable consumption^(75,76)^. Nonetheless, the literature on nutrition gamification for children and adolescents does not fully describe the content available in the apps, nor has there been an appraisal of their quality.

In this review, most apps focussed on promoting nutrition skills (i.e. cooking and baking). While there is strong evidence that involving children in food preparation and providing them with culinary or cooking skills can predict healthy eating or positive attitudes towards healthy eating, these studies focus on in-person engagement and not on examining the role of gamification in a child’s nutrition practices and health^(77,78)^. Due to limited research, it is unclear whether gamification centered on promoting nutrition skills among children is effective in promoting healthy nutrition practices. Nonetheless, there is a need for more nutrition-based app development with a diversity of content to improve not only cooking skills but also nutrition knowledge, attitudes and practices.

Regarding the mobile app quality ratings, while there is still room for improvement, the engagement, functionality and aesthetics mean sub-scores indicate that, on average, the apps were easy to learn and navigate, had a logical flow and visual appeal and were stylistically consistent. However, these apps did not contain evidence-based, credible nutrition information and were mainly skill-based instead of knowledge-based. In a recent study, the MARS was used to evaluate the quality of nutrition-related mobile apps available in China, albeit apps providing dietary guidance to adults. Similar to our study, the majority of apps were designed to assist with cooking, and the information quality score was the lowest among the four subsections of the MARS^(79)^.

The development of nutrition-related apps to improve nutrition practices among children and adolescents is noteworthy; however, the literature and this study point to the need for more robust approaches to app development. The development process should involve a multidisciplinary team of nutritionists, child health and development experts and graphic designers engaged in human-centered design, which heightens the focus on the needs of the end-user and involves children and adolescents in the intervention development process^(80)^.

### Strengths and limitations

This review has many strengths and potential limitations that need consideration. The appraisal method is subject to selection bias for a few main reasons, which may limit the generalisability of our findings. We only rated apps suitable for the iPhone, which can limit the generalisability of results for apps found in other mobile operating systems like Android. We only rated apps with content in English; hence, our results are only generalisable to apps in English. Next, the raters for the appraisal are adults, whereas the apps were intended for children as end users. Even though adult caregivers are most likely to choose apps, especially for younger children, adults and children may perceive quality differently. Ideally, we could include children appraisers, as the apps are designed for them; however, developing a child-led appraisal strategy would be challenging since we would need to accommodate varying developmental stages and age groups.

Another limitation was that any apps that included inappropriate content, such as references to drug or alcohol use, profanity or sexually explicit materials, were excluded. However, because these apps were not selected for instalment, it was difficult to assess why they were flagged as containing explicit material. For example, the app could have used alcohol as an example of beverages that should not be consumed, but we did not include it because it was deemed inappropriate.

Finally, although both reviewers (ES: iOS 7 and IB: iOS 6s) used iPhones for this review, the phones used by each reviewer were different. Although there is not much difference between iOS 6s and 7 devices, and thus the anticipated impact of using different phones was minimal, we acknowledge the possible effect this could have on the rating of the apps.

## Conclusion

Nutrition-related smartphone apps designed specifically for children and adolescents could serve as helpful tools to promote healthy nutrition and consequently prevent obesity. However, there are few free nutrition apps widely accessible for engagement. Although the apps in this review show promise in providing an engaging experience, this review highlights the need to develop apps with evidence-based nutrition education content that is age-appropriate for children and adolescents and evaluate their effectiveness on health outcomes. We propose accelerating the development of theory-guided apps using a human-centered and co-design approach with children for whom the apps are designed, with evidence-based nutrition content and evaluating the effectiveness on health outcomes.

## Supplementary material

For supplementary material accompanying this paper visit https://doi.org/10.1017/S1368980023000526. click here to view supplementary material
