# Supplementary material for: A systematic appraisal of the information, engagement, aesthetic and functional quality of nutrition-related smartphone apps for children and adolescents
Source: Public Health Nutr. 2023 Mar 13;26(7):1368–79. doi: 10.1017/S1368980023000526 (PMC10346071; doi:10.1017/S1368980023000526)
Supplement: Supplementary file 1 [file S1368980023000526sup.zip › S1368980023000526sup001.docx]

**Supplemental Table. Food Categories Represented in Each App Based on the *What We Eat in America Food Categories: 2017-2018***

| App Name | Milk & Dairy | Protein Foods | Mixed Dishes | Grains | Snacks & Sweets | Fruits | Vegetables | Beverages | Water | Fats & Oils | Condiments & Sauces | Sugars |
| --- | --- | --- | --- | --- | --- | --- | --- | --- | --- | --- | --- | --- |
| Baked Lasagna Chef Kids Cooking Game^37^ | X | X | X | X |  | X | X |  |  | X | X |  |
| Chef Cooking – Baby Cotton Candy Cooking Making & Dessert Make Games for Kids^38^ | X | X | X | X |  | X | X | X |  |  | X |  |
| Cooking Breakfast Maker^39^ | X | X |  | X |  |  | X | X |  | X | X |  |
| Cooking Court Food Fever^40^ | X | X | X | X | X |  | X | X |  |  | X |  |
| Cooking Girl, Amy and Cooking Kids Game^41^ |  | X |  |  |  |  | X |  |  | X | X |  |
| Cooking Time 2 – Sushi Make & Preschool Kids Game^42^ |  | X | X | X |  |  | X |  |  |  | X |  |
| Feed Twip^43^ | X | X |  | X | X | X | X | X | X | X |  | X |
| FoodLeap^44^ |  | X |  | X |  | X | X |  |  |  | X |  |
| Fresh Salad Bar: Healthy Green Food Making Game for Education & Learning^45^ |  |  |  |  |  | X | X |  |  |  |  |  |
| Health and Nutrition Quiz for Kids^46^ | X | X |  | X | X | X | X | X | X | X |  | X |
| Hot Soup Maker – Crazy Chef with Health Food Kitchen Adventure Spicy Cooking Fever^47^ |  | X | X |  |  |  | X |  |  |  |  |  |
| Ice Candy Fever Cooking Game – Cool Kids Food Chef^48^ |  |  |  |  |  | X |  |  | X |  |  |  |
| Ice Coffee Maker- Make Creamy Dessert in this Cooking Fever Game for kids^49^ |  |  |  |  |  |  |  | X | X |  |  |  |
| Kid Cooking Food: The Funny Restaurant Simulator Free Games^50^ | X | X | X | X |  | X | X | X |  |  | X |  |
| Kitchen Kids Cooking Chef: Let’s Cook the most Delicious Food^51^ |  | X |  |  |  | X | X |  |  | X |  |  |
| Mr J Cooks Food, Free Cooking Kids Game^52^ |  | X |  |  |  |  | X |  |  | X |  |  |
| Noodle Maker – Crazy Cooking Adventure for Little Kids Chef Master^53^ |  | X | X | X |  |  | X |  |  |  | X |  |
| Pasta Maker Kids Cook – Free Crazy Star Chef Adventure Girls Kitchen Cooking Games^54^ |  | X | X | X |  | X | X |  |  |  | X |  |
| Pasta Maker – Kitchen Cooking Chef and Fast Food Game^55^ |  |  | X | X |  |  | X |  |  | X | X |  |
| Sky Burger Maker Cooking Fever – Kids Games^56^ | X | X | X | X |  |  | X | X |  |  | X |  |
| Spaghetti Maker – Little Kids Cook Chinese Food in this Cooking Fever Game^57^ | X |  | X | X |  |  | X | X |  | X | X |  |
| Street Food Cooking Mania – Fun Kitchen Management^58^ | X | X | X | X |  | X | X | X |  | X | X |  |
| Veggie Bottoms^59^ |  | X |  |  |  | X | X |  |  |  |  |  |
| Zucchini Spaghetti Bolognese- Vegan Cooking Recipe with Emma: Game for Kids^60^ |  | X | X |  |  |  | X | X |  | X | X | X |
| N (%) of apps with food/drink in each category | **10**  **(42)** | **19**  **(79)** | **13**  **(54)** | **15**  **(63)** | **3**  **(13)** | **12**  **(50)** | **22**  **(92)** | **11**  **(46)** | **4**  **(17)** | **11**  **(46)** | **15**  **(63)** | **3**  **(13)** |
| If an “X” is marked in one of the 12 *What We Eat in America Food Categories*, this indicates that one or more of the foods/beverages included in that category were represented in the app.  Copyright ©Stoyan R Stoyanov, Leanne Hides, David J Kavanagh, Oksana Zelenko, Dian Tjondronegoro, Madhavan Mani. Originally published in JMIR Mhealth and Uhealth (http://mhealth.jmir.org), 11.03.2015. | | | | | | | | | | | | |
