# Supplementary material for: A systematic appraisal of the information, engagement, aesthetic and functional quality of nutrition-related smartphone apps for children and adolescents
Source: Public Health Nutr. 2023 Mar 13;26(7):1368–79. doi: 10.1017/S1368980023000526 (PMC10346071; doi:10.1017/S1368980023000526)
Supplement: Supplementary file 1 [file S1368980023000526sup.zip › S1368980023000526sup002.docx]

**Supplemental Methods**

**Supplemental Methods 1: Top Food Sources of Added Sugars, Saturated Fat, and Sodium**

- Top sources of food for added sugars

- Sweetened beverages (non-diet soft drinks, fruit drinks, flavored milk, and sports and energy drinks)
- Sweetened bakery products (cakes, pies, cookies, brownies, donuts, sweet rolls, and pastries)
- Other desserts (ice cream, frozen dairy desserts, puddings, gelatins, ices, and sorbets)
- Candy (those containing and not containing chocolate)
- Tea and coffee with added sugar
- Ready-to-eat cereals

-Top sources of food for saturated fat

- High fat meats (sausage, bacon, hot dogs, and ground beef that is less than 93% lean)
- Sandwiches (burritos, tacos, chicken/turkey, burgers, breakfast sandwiches, hotdogs, and other sandwiches)
- Sweetened bakery products (cakes, pies, cookies, brownies, donuts, sweet rolls, and pastries)
- Other desserts (ice cream, frozen dairy desserts, puddings, gelatins, ices, and sorbets)
- Rice, pasta, and other grain based mixed dishes
- Higher fat milk and yogurt
- Pizza
- Fried potatoes (French fries, tater tots, and hash browns)
- Chips, buttered popcorn, and ritz crackers

-Top sources of food for sodium

- Sandwiches (burritos, tacos, chicken/turkey, burgers, breakfast sandwiches, hotdogs, PBJ, and other sandwiches)
- Grain or Meat/Poultry/Fish-Mixed
- Dishes (mixed dishes, pasta/macaroni dishes, Mexican items, Indian/Asian items, and rice/rice dishes)
- Pizza
- Fried potatoes (French fries, tater tots, and hash browns)

**Supplemental Methods 2: Adapted Mobile App Rating Scale (MARS) Guide**

Adaptations/additions to the MARS response criteria, intended to improve interrater reliability, are highlighted in **bold** below.

App Objective Quality Ratings

Section A. Engagement – fun, interesting, customizable, interactive (e.g., sends alerts, messages, reminders, feedback, enables sharing), well-targeted to audience

1. Entertainment: Is the app fun/entertaining to use? Does it use any strategies to increase engagement through entertainment (e.g., through gamification)?

**How much time would you spend using the app? Is it entertaining for kids?**

1. Dull, not fun or entertaining at all **(<1 minute; turn off app)**

2. Mostly boring **(no gamification present but still slightly entertaining)**

3. Ok, fun enough to entertain for a brief time (**>1 minute but** < 5 minutes)

4. Moderately fun and entertaining, would entertain user for some time (5-10 minutes total)

5. Highly entertaining and fun, would stimulate repeat use

1. Interest: Is the app interesting to use? Does it use any strategies to increase engagement by presenting its content in an interesting way?

**Does the app present content in an interesting way?**

1. Not interesting at all **(<1 minute; turn off app)**

2. Moderately uninteresting

3. OK, neither interesting nor uninteresting; would engage user for a brief time (**>1 minute but** < 5 minutes)

4. Moderately interesting; would engage user for some time (5-10 minutes total)

5. Very interesting, would engage user in repeat use

1. Customization: Does it provide/retain all necessary settings/preferences for apps features (e.g., sound, content, notifications, etc.)?

**Does the app allow you to make customizations and if so, do the customizations remain after logging out/closing app?**

1. Does not allow any customization or requires setting to be input every time

2. Allows insufficient customization limiting functions **(e.g., multiple profiles not allowed when it would be needed; some customizations but limits use somehow; one customization but may or may not remain after logging out/closing app)**

3. Allows basic customization to function adequately **(e.g., have audio adjustments; 2 customizations but may or may not remain after logging out/closing app)**

4. Allows numerous options for customization **(but settings may not remain after logging out/closing app)**

5. Allows complete tailoring to the individual’s characteristics/preferences, retains all settings

1. Interactivity: Does it allow user input, provide feedback, contain prompts (reminders, sharing options, notifications, etc.)? Note: these functions need to be customizable and not overwhelming in order to be perfect.

**Positive feedback alone or with goal in mind is considered feedback/interactivity.**

1. No interactive features and/or no response to user interaction

2. Insufficient interactivity, or feedback, or user input options, limiting functions

3. Basic interactive features to function adequately **(i.e., positive reinforcement to let users know they are progressing through the app correctly)**

4. Offers a variety of interactive features/feedback/user input options **(i.e., specific feedback rather than just positive reinforcement)**

5. Very high level of responsiveness through interactive features/feedback/user input options

1. Target group: Is the app content (visual information, language, design) appropriate for your target audience?

**Based on the reviewer perceived age for the app (readability, design, and content).**

1. Completely inappropriate/unclear/confusing

2. Mostly inappropriate/unclear/confusing

3. Acceptable but not targeted. May be inappropriate/unclear/confusing

4. Well-targeted, with negligible issues **(e.g., some issues might be using words like “fat” or use of some medical jargon)**

5. Perfectly targeted, no issues found

Section B. Functionality – app functioning, easy to learn, navigation, flow logic, and gestural design of app

1. Performance: How accurately/fast do the app features (functions) and components (buttons/menus) work?

**Is the app working as it should? Do the movements you do consistently give what you**

**want?**

1. App is broken; no/insufficient/inaccurate response (e.g., crashes/bugs/broken features, etc.)

2. Some functions work, but lagging or contains major technical problems **(major issues, not all the functions work)**

3. App works overall. Some technical problems need fixing/Slow at times **(the few technical problems encroach on the functionality of the app/there are a few glitches that ruin the app experience)**

4. Mostly functional with minor/negligible problems **(small glitches that do not encroach on the experience, e.g., being asked to drag when you are supposed to tap)**

5. Perfect/timely response; no technical bugs found/contains a ‘loading time left’ indicator

1. Ease of use: How easy is it to learn how to use the app; how clear are the menu labels/icons and instructions?

1. No/limited instructions; menu labels/icons are confusing; complicated **(too complicated you can’t use it/don’t know what to do even after significant effort)**

2. Useable after a lot of time/effort **(if spending > 5 mins to figure out)**

3. Useable after some time/effort **(if spending < 5 mins to figure out)**

4. Easy to learn how to use the app (or has clear instructions)

5. Able to use app immediately; intuitive; simple

1. Navigation: Is moving between screens logical/accurate/appropriate/ uninterrupted; are all necessary screen links present?

1. Different sections within the app seem logically disconnected and random/confusing/ navigation is difficult

2. Usable after a lot of time/effort **(multiple ads in a row that greatly interrupt the experience)**

3. Usable after some time/effort **(an ad that stops you from progressing from page to page as you would like, can’t move onto the next screen without watching an ad)**

4. Easy to use or missing a negligible link **(no return button to home screen or back button)**

5. Perfectly logical, easy, clear and intuitive screen flow throughout, or offers shortcuts

1. Gestural design: Are interactions (taps/swipes/pinches/scrolls) consistent and intuitive across all components/screens?

1. Completely inconsistent/confusing

2. Often inconsistent/confusing **(there are multiple inconsistencies)**

3. OK with some inconsistencies/confusing elements **(2 inconsistencies)**

4. Mostly consistent/intuitive with negligible problems **(1 inconsistency)**

5. Perfectly consistent and intuitive

Section C. Aesthetics – graphic design, overall visual appeal, color scheme, and stylistic consistency

1. Layout: Is arrangement and size of buttons/icons/menus/content on the screen appropriate or zoomable if needed?

**Focused on the screen layout. Are there pop ups or ads that make it difficult to see**

**things on the screen?**

1. Very bad design, cluttered, some options impossible to select/locate/see/read device display not optimized **(layout makes it impossible to use)**

2. Bad design, random, unclear, some options difficult to select/locate/see/read **(layout makes it difficult to use)**

3. Satisfactory, few problems with selecting/locating/seeing/reading items or with minor screen-size problems **(the layout has > 2 issues or the issues are starting to impede on the function of the app)**

4. Mostly clear, able to select/locate/see/read items (**the layout has 1-2 issues, but you are still able to use the app)**

5. Professional, simple, clear, orderly, logically organized, device display optimized. Every design component has a purpose

1. Graphics: How high is the quality/resolution of graphics used for buttons/icons/menus/ content?

1. Graphics appear amateur, very poor visual design -disproportionate, completely stylistically inconsistent

2. Low quality/low resolution graphics; low quality visual design –disproportionate, stylistically inconsistent **(e.g., stylistically inconsistent - some design elements are targeted at small children, but others are more targeted at teenagers; low quality design - can’t figure out what some of the food items are or components of the food don’t match what you are making, disproportionate sizing that stands out, strikingly different stylistically)**

3. Moderate quality graphics and visual design (generally consistent in style) **(inconsistencies** **aren’t striking or foods don’t look like they are supposed to,**

**but you can guess with context)**

4. High quality/resolution graphics and visual design –mostly proportionate, stylistically consistent **(very small inconsistency in graphics but it doesn’t**  **detract from you knowing what something is)**

5. Very high quality/resolution graphics and visual design -proportionate, stylistically consistent throughout

1. Visual appeal: How good does the app look?

1. No visual appeal, unpleasant to look at, poorly designed, clashing/mismatched colors **(no variation in color so that it’s really boring to look at or use of color interferes with ability to use the app)**

2. Little visual appeal –poorly designed, bad use of color, visually boring **(lots of bland colors/little variation)**

3. Some visual appeal –average, neither pleasant, nor unpleasant **(use of color**  **but neither visually appealing nor unappealing - lots of similar/bland colors but**  **use of color makes sense for the app)**

4. High level of visual appeal –seamless graphics –consistent and professionally designed **(multiple colors used, there is visual appeal, but app does not use color strategically)**

5. As above + very attractive, memorable, stands out; use of color enhances app features/menus **(use of color accentuates important parts of the app/color is used strategically)**

Section D. Information – Contains high quality information (e.g., text, feedback, measures, references) from a credible source. Select N/A if the app component is irrelevant.

1. Accuracy of app description (in app store): Does app contain what is described?

1. Misleading. App does not contain the described components/functions. Or has no description

2. Inaccurate. App contains very few of the described components/functions

3. OK. App contains some of the described components/functions

4. Accurate. App contains most of the described components/functions

5. Highly accurate description of the app components/functions

1. Goals: Does app have specific, measurable and achievable goals (specified in app store description or within the app itself)?

**Is the goal measurable/achievable? What is the desired outcome of the app (e.g.,**  **making a cup of coffee, making soup—this does not need to be stated explicitly as**  **long as it is obvious)?**

N/A. Description does not list goals, or app goals are irrelevant to research goal (e.g., using a game for educational purposes) **(no goals stated, just assembling food with no goal, one line description that doesn’t tell you much; no skill development, e.g., just feeding customers)**

1. App has no chance of achieving its stated goals **(no specific food item or lots of random ingredients so user won’t likely be able to make the foods on their own; ingredients don’t make up the food item)**

2. Description lists some goals, but app has very little chance of achieving them **(some goals are stated for a specific food type, for example making hot soup or coffee, but user probably wouldn’t be able to make on their own)**

3. OK. App has clear goals, which may be achievable **(goals could be achievable but they are hard to measure, e.g., assembly/skill development, such as knife skills)**

4. App has clearly specified goals, which are measurable and achievable **(goals are stated and could may be achievable on one’s own without the app but there are no directions, e.g., making a salad but without directions)**

5. App has specific and measurable goals, which are highly likely to be achieved **(specific, achievable, stated goals with written directions, feedback, or recipe in the app so the user would be able to make the food/apply the learned skills on their own)**

15. Quality of information: Is app content correct, well written, and relevant to the

goal/topic of the app?

**Where content refers to written or audio information to support food skills/literacy.**

N/A. There is no information within the app **(no written or audio content in the app)**

1. Irrelevant/inappropriate/incoherent/incorrect **(very incorrect information or harmful information, e.g., eating pills)**

2. Poor. Barely relevant/appropriate/coherent/may be incorrect **(largely irrelevant facts about the foods, e.g., food jokes)**

3. Moderately relevant/appropriate/coherent/and appears correct **(a generally 50/50 split of irrelevant and relevant facts about food)**

4. Relevant/appropriate/coherent/correct **(some jokes but mostly relevant information about food)**

5. Highly relevant, appropriate, coherent, and correct **(every written/audio information serves a learning purpose)**

16. Quantity of information: Is the extent coverage within the scope of the app; and

comprehensive but concise?

**Where content refers to written or audio information to support food skills/literacy.**

N/A. There is no information within the app **(no written or audio content in the app)**

1. Minimal or overwhelming **(e.g., overwhelming – content too much for targeted age group; minimal would be almost no audio or written content)**

2. Insufficient or possibly overwhelming **(insufficient or possibly overwhelming amount of information to achieve the goal, wouldn’t be able to achieve steps based on information, missing steps)**

3. OK but not comprehensive or concise **(could probably achieve the stated goal based on the written/audio information provided)**

4. Offers a broad range of information, has some gaps or unnecessary detail; or has no links to more information and resources **(some irrelevant information added in, no links to added resources, gaps in the details)**

5. Comprehensive and concise; contains links to more information and resources **(some really good information with available links to more information, comprehensive and concise)**

17. Visual information: Is visual explanation of concepts –through charts/graphs/images/

videos, etc. –clear, logical, correct?

**Focus on visual demonstrations of skills, graphs, and pictures of food.**

N/A. There is no visual information within the app **(app only contains audio or text)**

1. Completely unclear/confusing/wrong or necessary but missing **(can’t tell what any of the food images are, can’t interpret graphs, skills are incorrect or unsafe)**

2. Mostly unclear/confusing/wrong **(can’t distinguish most of the ingredients, missing multiple steps of a skill)**

3. OK but often unclear/confusing/wrong **(process doesn’t seem completely accurate, missing steps of a skill, can’t distinguish some ingredients)**

4. Mostly clear/logical/correct with negligible issues **(can tell what foods are based on context)**

5. Perfectly clear/logical/correct

18. Credibility: Does the app come from a legitimate source (specified in app store

description or within the app itself)?

1. Source identified but legitimacy/trustworthiness of source is questionable (e.g., commercial business with vested interest)

2. Appears to come from a legitimate source, but it cannot be verified (e.g., has no webpage)

3. Developed by small NGO/institution (hospital/centre, etc.) /specialized commercial business, funding body

4. Developed by government, university or as above but larger in scale

5. Developed using nationally competitive government or research funding (e.g., Australian Research Council, NHMRC)

19. Evidence base: Has the app been trialled/tested; must be verified by evidence (in

published scientific literature)?

N/A. The app has not been trialled/tested

1. The evidence suggests the app does not work

2. App has been trialled (e.g., acceptability, usability, satisfaction ratings) and has partially positive outcomes in studies that are not randomized controlled trials (RCTs), or there is little or no contradictory evidence.

3. App has been trialled (e.g., acceptability, usability, satisfaction ratings) and has positive outcomes in studies that are not RCTs, and there is no contradictory evidence.

4. App has been trialled and outcome tested in 1-2 RCTs indicating positive results

5. App has been trialled and outcome tested in >3 high quality RCTs indicating positive results

App Subjective Quality Ratings

Section E.

20. Would you recommend this app to people who might benefit from it?

1. Not at all: I would not recommend this app to anyone

2. There are very few people I would recommend this app to

3. Maybe: There are several people whom I would recommend it to

4. There are many people I would recommend this app to

5. Definitely: I would recommend this app to everyone

21. How many times do you think you would use this app in the next 12 months if it was

relevant to you?

1. None **(only one level to the game so it would be boring to replay, no variation)**

2. 1-2 **(multiple levels to get through so user can’t play all at once but the game itself is boring)**

3. 3-10 **(variation in what you can do or make; might use infrequently but there is a specific reason to go back and use it)**

4. 10-50 **(multiple levels and entertaining, fits into nighttime routine or daily life patterns)**

5. >50 **(incentives to go back to the app, e.g., ability to track daily progress, or the app changes over time)**

22. Would you pay for this app?

1. No

3. Maybe

5. Yes

23. What is your overall star rating of this app?

1. * One of the worst apps I’ve used

2. **

3. *** Average

4. ****

5. ***** One of the best apps I’ve used

24. Do you feel this app was appropriate for the age group identified?

1. Not at all

3. Maybe **(its content is potentially too challenging for example due to jargon)**

5. Definitely
